# Maintenance of Custom-Made Subperiosteal Implants: A Narrative Review of Indirect Evidence and Preliminary Clinical Considerations

**DOI:** 10.3390/jcm15114333

**Published:** 2026-06-03

**Authors:** Valentina Dessì, Luigi Angelo Vaira

**Affiliations:** 1 Private Practice, 07100 Sassari, Italy; 2 Maxillo-Facial Surgery Operative Unit, Department of Medicine, Surgery and Pharmacy, University of Sassari, 07100 Sassari, Italy

**Keywords:** subperiosteal implants, custom-made implants, implant maintenance, peri-implant care, oral hygiene, prosthetic design, risk-based follow-up, supportive peri-implant therapy

## Abstract

**Background:** Custom-made subperiosteal implants have re-emerged as a valuable option for the rehabilitation of patients with severe maxillofacial atrophy and post-oncological defects. Despite advances in digital workflows and implant design, their unique anatomical, biological, and prosthetic characteristics pose specific challenges for long-term maintenance, and no dedicated standardized guidelines are currently available. **Methods:** This narrative review critically appraises the available literature on implant maintenance and related fields. A comprehensive search was conducted across PubMed, Scopus, and Web of Science, including studies on peri-implant maintenance, supportive periodontal therapy, full-arch and zygomatic implant rehabilitations, and subperiosteal implants. Due to the lack of direct evidence, a qualitative narrative synthesis was adopted to develop preliminary clinical considerations for maintenance of custom-made subperiosteal implants. These considerations should be interpreted as an expert-informed perspective rather than validated clinical guidelines. **Results:** Conventional maintenance protocols developed for endosseous implants are not directly transferable to subperiosteal implants due to differences in the implant–tissue interface, biomechanics, diagnostic parameters, and hygiene accessibility. Key challenges include the absence of a conventional peri-implant sulcus, possible implant exposure, complex prosthetic geometries, and potential susceptibility to biofilm accumulation in areas with limited access. Evidence from related fields highlights the importance of structured maintenance, individualized risk-based follow-up, effective biofilm control, and patient-specific home-care strategies. **Conclusions:** Preliminary evidence-informed clinical considerations for the maintenance of subperiosteal implants are proposed, with emphasis on plaque control, individualized follow-up, descriptive clinical monitoring, and hygiene-oriented prosthetic and surgical planning. These considerations are not intended as validated guidelines, but as a practical starting point for clinical reasoning in an area where dedicated evidence remains limited.

## 1. Introduction

In recent years, the reintroduction of custom-made subperiosteal implants has provided a valuable therapeutic option for the rehabilitation of patients with severe maxillary [1,2,3] or mandibular atrophy [4,5,6,7], as well as for those presenting with complex post-oncological defects [8,9,10,11]. Advances in digital workflows, including computer-aided design and manufacturing (CAD/CAM) technologies, have significantly improved the fit, stability, and clinical applicability of these patient-specific devices, leading to renewed clinical interest after decades of limited use associated with earlier implant designs [12,13].

Historically, subperiosteal implants were associated with a high rate of complications, including chronic inflammation, exposure, and infection, largely attributed to poor adaptation, inadequate fixation, and the absence of osseointegration [14,15,16]. Contemporary designs, however, differ substantially from their predecessors, relying on rigid fixation, improved biomaterials, and precise anatomical adaptation, thereby overcoming many of the limitations reported in early clinical experiences. Nevertheless, despite these technological advancements, the biological behavior of subperiosteal implants remains fundamentally distinct from that of conventional endosseous implants.

Unlike endosseous implants, subperiosteal implants are positioned over the cortical bone surface and include multiple transmucosal components. These features create specific challenges related to soft tissue stability, hygiene accessibility, and clinical monitoring, which require dedicated discussion and cautious interpretation [17,18,19,20].

Maintenance therapy is widely recognized as a critical determinant of long-term success in implant dentistry. A substantial body of evidence has demonstrated that supportive peri-implant therapy significantly reduces the incidence of peri-implant diseases and implant loss in patients rehabilitated with endosseous implants [21,22,23,24,25]. Regular professional debridement, patient-specific oral hygiene instructions, and risk-based recall intervals are considered essential components of contemporary implant care [22,24,26,27]. However, these protocols have been developed and validated almost exclusively in the context of osseointegrated implants.

At present, there is a clear lack of standardized guidelines or evidence-based protocols specifically addressing the maintenance of patients rehabilitated with subperiosteal implants. While the importance of maintenance therapy is consistently emphasized across the literature, detailed and structured indications on how such maintenance should be performed in this specific clinical context are largely lacking. The available studies are predominantly limited to case reports, technical notes, and short-term case series, with minimal focus on long-term maintenance strategies or clearly defined hygiene protocols. As a result, clinicians are often required to extrapolate principles derived from conventional implantology, despite the significant biological and structural differences between these implant systems.

Given these considerations, a critical narrative synthesis of the available direct and indirect evidence may help clarify which maintenance principles can be cautiously considered in this field. Because direct clinical evidence is scarce, the contribution of the present article is not the validation of a maintenance protocol, but the organization of fragmented evidence and clinical reasoning into a transparent, preliminary structure that may guide future research and consensus development.

Therefore, the aim of the present narrative review is twofold: first, to summarize the available direct and indirect evidence relevant to the maintenance of custom-made subperiosteal implants; and second, to propose preliminary evidence-informed clinical considerations that may help clinicians structure follow-up and hygiene management in this emerging field. These considerations should not be interpreted as validated guidelines, but rather as an expert-informed clinical perspective requiring future validation.

## 2. Materials and Methods

### 2.1. Study Design

The present study was conceived as a narrative review aimed at critically appraising the available literature on implant maintenance and related fields, with the objective of developing preliminary evidence-informed clinical considerations for patients rehabilitated with custom-made subperiosteal implants.

In light of the limited number of studies specifically addressing subperiosteal implants and the absence of high-level evidence in this area, a narrative approach was considered the most appropriate methodology. This design allowed the integration of heterogeneous sources of evidence, including clinical studies, reviews, and technically oriented reports, as well as the incorporation of clinically relevant considerations derived from related disciplines.

This review was not designed as a systematic review and does not aim to provide evidence-based clinical guidelines. No formal risk-of-bias assessment, certainty-of-evidence grading, or expert consensus procedure was performed. Therefore, the proposed clinical considerations should be interpreted as an expert-informed synthesis intended to support clinical reasoning in an area where direct evidence is currently scarce. The strength of each statement is limited by the indirectness and heterogeneity of the available evidence.

### 2.2. Search Strategy

A comprehensive literature search was conducted across the electronic databases PubMed, Scopus, and Web of Science. The search strategy combined Medical Subject Headings (MeSH) and free-text terms related to subperiosteal implants and implant maintenance (Supplementary Table S1). Particular attention was given to capturing both historical and contemporary terminology, including variations such as “juxtaosseous implants” and “custom-made implants.” These terms were combined with keywords related to maintenance, including “peri-implant maintenance,” “supportive therapy,” “oral hygiene,” and “recall program.” To ensure comprehensive coverage of the topic, additional searches were performed using broader terms related to implant dentistry, full-arch rehabilitation, and supportive periodontal therapy. Reference lists of selected articles were also screened to identify further relevant publications.

Because this was a narrative review, the search strategy was intended to be broad and clinically oriented rather than systematic. The search aimed to identify representative and clinically relevant evidence, including systematic reviews, scoping reviews, cohort studies, case series, technical reports, and consensus documents. However, the possibility that relevant publications were not identified or included cannot be excluded. This limitation has been explicitly acknowledged.

### 2.3. Eligibility Criteria

Studies were considered eligible if they addressed implant maintenance or provided information potentially applicable to the management of subperiosteal implants. Particular emphasis was placed on clinical studies investigating peri-implant maintenance, including randomized controlled trials, cohort studies, and case–control studies, as well as systematic and narrative reviews focusing on supportive implant therapy. Additional sources included studies on full-arch implant rehabilitation and complex implant-supported prostheses, as well as investigations exploring biofilm control and professional oral hygiene around implants. Given the scarcity of direct evidence, articles specifically describing subperiosteal implants, including case series and technical reports, were also included when relevant to the topic. Studies not related to implant maintenance or oral hygiene were excluded, as were in vitro investigations lacking clear clinical implications. Articles not available in English and publications without accessible full text were also excluded.

### 2.4. Study Selection and Data Extraction

The selection process was conducted through an initial screening of titles and abstracts to identify potentially relevant studies, followed by full-text evaluation of eligible articles. Although a formal PRISMA-style selection process was not performed, an approximate descriptive record of the selection process was maintained. Overall, 312 records were identified across database searches and reference-list screening. After removal of duplicates and clearly irrelevant records, 178 titles and abstracts were screened, 111 full-text articles were assessed for relevance, and 95 publications were ultimately included in the narrative synthesis. These figures should be interpreted as descriptive estimates rather than as a formal PRISMA-based selection flow. Given the narrative nature of the review, no formal risk of bias assessment or quantitative synthesis was performed. No PRISMA flow diagram was generated, and no formal certainty-of-evidence assessment was conducted. Therefore, the selection process should not be interpreted as equivalent to that of a systematic review. Instead, studies were selected based on their clinical relevance, consistency with the review objectives, and contribution to the understanding of maintenance strategies in implant dentistry and related fields.

From the included studies, relevant information was extracted regarding maintenance protocols and recall intervals, professional hygiene techniques and instruments, patient compliance and associated risk factors, as well as outcomes related to peri-implant health and implant survival.

### 2.5. Data Synthesis and Development of Clinical Considerations

Due to the heterogeneity of the available literature and the lack of direct evidence specifically addressing subperiosteal implant maintenance, a qualitative narrative synthesis was adopted. Evidence derived from endosseous implantology, supportive periodontal therapy, zygomatic implant rehabilitation, full-arch prosthetic maintenance, and reconstructive surgery was critically interpreted in light of the unique biological and structural characteristics of subperiosteal implants. Based on this process, preliminary clinical considerations were formulated. These considerations should not be interpreted as a standardized protocol or as evidence-based recommendations, but rather as non-validated guidance principles to be adapted to individual patient needs, prosthetic design, hygiene accessibility, clinical findings, and patient-related risk factors.

## 3. Biological and Structural Peculiarities of Subperiosteal Implants

### 3.1. Anatomical and Biomechanical Characteristics

Custom-made subperiosteal implants are characterized by a patient-specific framework designed to rest directly on the cortical bone surface, typically extending over large anatomical areas of the maxilla or mandible [28,29,30,31]. Unlike endosseous implants, which are inserted within the bone and achieve primary stability through mechanical engagement followed by osseointegration, subperiosteal implants rely on a combination of anatomical adaptation and rigid fixation, often achieved through osteosynthesis screws [32,33].

The introduction of digital workflows and CAD/CAM manufacturing has significantly improved the precision of implant fit and load distribution, reducing micromovements and enhancing mechanical stability [1,2,3,12,13]. Finite element analyses have suggested that modern subperiosteal implants can achieve favorable stress distribution across the supporting bone, comparable to other reconstructive solutions when properly designed and fixed. Nevertheless, load transmission occurs predominantly at the bone surface rather than through a bone–implant interface, resulting in a fundamentally different biomechanical environment [34,35,36,37,38].

These biomechanical differences may have implications for long-term mechanical stability and tissue response under functional loading, although their clinical significance remains insufficiently defined.

### 3.2. Soft Tissue Interface and Transmucosal Components

A key distinguishing feature of subperiosteal implants is the presence of multiple transmucosal abutments emerging through the oral mucosa. These components create a direct communication between the oral environment and the underlying implant framework. In contrast to endosseous implants, where a structured peri-implant soft tissue seal can develop around the transmucosal portion, subperiosteal implants may not benefit from a comparable circumferential biological barrier. The soft tissue interface is likely to differ from that observed around conventional endosseous implants [39,40], although its histological and functional characteristics have not been adequately investigated in contemporary subperiosteal implant systems. Moreover, the presence of multiple transmucosal passages increases the overall surface area exposed to the oral cavity, potentially amplifying the risk of microbial ingress. The quality and thickness of the peri-implant mucosa, which may already be compromised in patients with severe atrophy or previous surgical interventions, further influence the stability of this interface [41,42,43].

Partial exposure of subperiosteal implant components has been reported in contemporary clinical series, with variable prevalence and clinical relevance [41,44,45,46,47]. Although limited exposures may remain asymptomatic and may sometimes be managed conservatively, exposed metallic surfaces may represent plaque-retentive areas and should be documented during follow-up. The prognostic significance of asymptomatic exposure, however, remains uncertain and requires dedicated longitudinal investigation.

### 3.3. Biofilm Accumulation, Microbial Ecology, and Host Response

The structural complexity of subperiosteal implants, combined with their extensive surface area and limited accessibility, may create plaque-retentive areas, particularly around irregular geometries, undercuts, and junctional zones between the implant framework, transmucosal components, and soft tissues [48]. In endosseous implantology, peri-implant diseases are increasingly interpreted as the result of a complex interaction between dysbiotic biofilm communities, host immune response, local tissue conditions, and implant-related factors rather than as a purely linear consequence of bacterial accumulation [49,50,51,52,53,54]. Recent studies and reviews have highlighted that peri-implantitis may be associated with shifts in microbial diversity and composition, immune dysregulation, inflammatory cytokine activity, and bidirectional host–microbiome interactions [51,52,53].

In the context of subperiosteal implants, these mechanisms remain largely unexplored. Nevertheless, the presence of broad implant–soft tissue interfaces, multiple transmucosal components, exposed or partially exposed metallic surfaces, and complex subprosthetic geometries may plausibly influence local biofilm retention and host response. These considerations may be particularly relevant in medically complex or post-oncologic patients, in whom radiotherapy, xerostomia, immunosuppression, diabetes, smoking, or reduced soft tissue quality may further alter mucosal defense and inflammatory control. However, direct studies evaluating biofilm ecology, microbial burden, inflammatory mediators, or disease progression around contemporary subperiosteal implants are lacking. Therefore, the microbiological and inflammatory considerations discussed here should be interpreted as biologically plausible hypotheses rather than established evidence specific to this implant system.

### 3.4. Clinical Implications of Structural and Biological Differences

The anatomical and biological characteristics described above indicate that conventional peri-implant maintenance protocols cannot be directly transferred to subperiosteal implants. In particular, the absence of a conventional circumferential peri-implant sulcus, the presence of multiple transmucosal components, possible implant exposure, and variable prosthetic accessibility require a different approach to clinical monitoring and maintenance.

Rather than relying primarily on probing depth or marginal bone-level changes, follow-up should focus on descriptive clinical parameters, including soft tissue condition, presence and progression of exposure, suppuration, prosthetic cleanability, plaque accumulation, and patient-reported symptoms. These aspects are discussed in detail in the clinical monitoring and maintenance sections below.

## 4. Indirect Evidence and Conceptual Reference Models from Related Fields

### 4.1. Peri-Implant Maintenance in Endosseous Implants

Maintenance therapy represents a cornerstone in contemporary implant dentistry and is widely recognized as a critical factor for the long-term success of osseointegrated implants. A substantial body of evidence has demonstrated that regular supportive peri-implant therapy significantly reduces the incidence of peri-implant diseases and implant loss [22,23,24,25].

Long-term studies have shown that patients enrolled in structured maintenance programs exhibit lower rates of peri-implantitis and improved implant survival compared to those with irregular follow-up [23,24,55]. Key components of these programs include periodic professional debridement, reinforcement of oral hygiene instructions, and risk-based recall intervals tailored to individual patient profiles.

Professional hygiene protocols commonly involve non-invasive or implant-specific instrumentation, including air-polishing devices with low-abrasive powders (e.g., glycine or erythritol), which have been shown to be effective in disrupting biofilm while preserving implant surfaces [21,56]. Dedicated ultrasonic tips designed for implant maintenance may also be considered when access is limited and mechanical debridement is required. In addition, personalized home-care instructions represent a fundamental component of maintenance therapy, as patient compliance has been consistently identified as a major determinant of treatment outcomes, further emphasizing the importance of continuous motivation and education [24,25,27].

Despite the robustness of this evidence, it is important to underline that these protocols have been developed specifically for endosseous implants, characterized by a relatively confined transmucosal interface and the presence of a peri-implant soft tissue seal. Accordingly, these data should not be considered directly applicable to subperiosteal implants. They are used here only to identify general principles of maintenance care, such as biofilm control, patient compliance, and structured follow-up, which require specific adaptation before being considered in the context of subperiosteal implant rehabilitation.

### 4.2. Maintenance in Full-Arch and Complex Implant Rehabilitations

Patients rehabilitated with full-arch implant-supported prostheses present additional challenges in terms of hygiene accessibility and biofilm control [20,54]. The presence of extensive prosthetic frameworks, reduced visibility, and limited access to critical areas often leads to the accumulation of plaque, calculus, and food debris, particularly in subprosthetic and posterior regions, thereby increasing the risk of peri-implant inflammation [49,57,58]. However, these principles should be translated cautiously, as subperiosteal implants present additional biological and anatomical differences that may further modify maintenance requirements.

Studies on full-arch rehabilitations have highlighted the importance of individualized hygiene protocols based on prosthetic accessibility, patient dexterity, and the ability to perform effective home care. Interdental brushes and soft-bristle toothbrushes are commonly used as home-care aids, whereas professional maintenance may include air-polishing and dedicated ultrasonic instrumentation when clinically indicated [59,60,61,62]. Regular professional maintenance may be required to compensate for the increased difficulty in achieving effective home care, although recall intervals should be adapted to the individual risk profile rather than predefined according to fixed schedules.

### 4.3. Maintenance in Zygomatic Implant Rehabilitation

Zygomatic implant rehabilitation represents a particularly relevant clinical model for understanding maintenance challenges in complex implant-supported prostheses. These implants are typically indicated in patients with severe maxillary atrophy or post-oncological defects and are characterized by unique anatomical and prosthetic configurations [63,64]. Unlike conventional endosseous implants, zygomatic implants often present reduced or absent crestal bone support in their coronal portion, with the transmucosal pathway predominantly surrounded by soft tissues. This results in a peri-implant environment that differs significantly from that of standard implants and shares important similarities with subperiosteal implant configurations.

Clinical evidence indicates that, although zygomatic implants exhibit high survival rates, they require structured and individualized maintenance strategies to support long-term success [65,66,67,68]. In particular, patients with a history of periodontitis or poor oral hygiene compliance tend to exhibit increased bacterial colonization and worse peri-implant clinical parameters, supporting the need for more frequent professional hygiene sessions [68]. Furthermore, biological complications such as soft tissue dehiscence, sinus-related conditions, and peri-implant inflammation have been consistently reported, highlighting the importance of continuous monitoring and early intervention [65,69].

In response to these challenges, specific maintenance approaches have been proposed, emphasizing structured follow-up programs, detailed clinical evaluation of transmucosal pathways, and intensive patient education [68]. These approaches often include systematic clinical assessments, closer monitoring during the early post-loading phase when indicated, and hygiene instructions tailored to the specific anatomical and prosthetic configuration.

### 4.4. Insights from Reconstructive and Oncologic Surgery

Additional insights can be derived from the management of patients undergoing maxillofacial reconstructive or oncologic surgery, where the presence of metallic hardware, altered anatomy, and compromised soft tissues may increase susceptibility to infection and complicate hygiene procedures [70,71,72].

In these clinical scenarios, preventive strategies typically rely on meticulous oral hygiene protocols, close clinical monitoring, and early intervention in the presence of tissue breakdown or infection [70,71,72]. The conservative management of exposed or partially exposed hardware through enhanced hygiene measures and local care is particularly relevant, as it closely parallels the clinical challenges observed in subperiosteal implant rehabilitations. Moreover, the frequent presence of comorbidities, previous radiotherapy, and reduced manual dexterity in these patients reinforces the need for simplified yet effective hygiene protocols and more intensive professional follow-up [72].

### 4.5. Supportive Periodontal Therapy as a Reference Model

Supportive periodontal therapy provides a well-established framework for long-term maintenance in patients with a history of periodontal disease [73,74,75]. Its principles, which include regular recall visits, professional biofilm removal, and risk-based patient management, have been successfully translated into implant dentistry. Evidence from periodontal literature has consistently demonstrated that structured maintenance programs significantly reduce disease recurrence and contribute to the long-term preservation of treatment outcomes [73,74]. Although originally developed for natural dentition, these concepts offer a valuable model for managing chronic inflammatory risks associated with implant-supported rehabilitations.

In the context of subperiosteal implants, where the potential for biofilm-related complications may be amplified, the adoption of a structured and proactive maintenance strategy appears particularly relevant.

### 4.6. Translational Considerations for Subperiosteal Implants

Taken together, the evidence from implant dentistry, periodontal maintenance, reconstructive surgery, and zygomatic implant rehabilitation provides conceptual reference points rather than directly transferable protocols for subperiosteal implant maintenance. The degree of indirectness remains substantial, because subperiosteal implants differ from these systems in terms of anatomical position, tissue interface, biomechanics, diagnostic parameters, and hygiene accessibility. Nevertheless, some broad principles—particularly biofilm control, prosthetic accessibility, patient compliance, and structured follow-up—may be clinically relevant if cautiously adapted to the specific characteristics of subperiosteal implants (Table 1).

The potential risks of over-translating concepts from biologically distinct implant systems should also be considered. First, diagnostic parameters validated for endosseous implants, such as probing depth or marginal bone-level changes, may lead to misleading interpretation when applied to subperiosteal implants, which lack a circumferential endosseous interface. Second, maintenance strategies developed for conventional implant prostheses may underestimate the relevance of framework exposure, broad soft-tissue contact areas, and complex subprosthetic geometries. Third, recall intervals derived from supportive periodontal or peri-implant therapy may not adequately reflect the risk profile of patients treated with subperiosteal implants, particularly in cases involving oncologic defects, previous radiotherapy, reduced soft-tissue quality, or limited manual dexterity. Therefore, indirect evidence should be used only to identify general principles, while clinical decisions should remain individualized and guided by the specific anatomical, prosthetic, and patient-related characteristics of each rehabilitation.

Based on these considerations, the following section outlines preliminary clinical considerations intended to support maintenance planning without implying validated or directly transferable recommendations.

## 5. Preliminary Clinical Considerations for Maintenance of Subperiosteal Implants

### 5.1. Rationale and General Principles

Based on the indirect evidence summarized above, the following preliminary clinical considerations are proposed to support maintenance planning for custom-made subperiosteal implants. They are intended as non-validated guidance principles and should be adapted to each patient according to prosthetic design, hygiene accessibility, tissue conditions, clinical stability, and individual risk profile.

**Figure 1:**
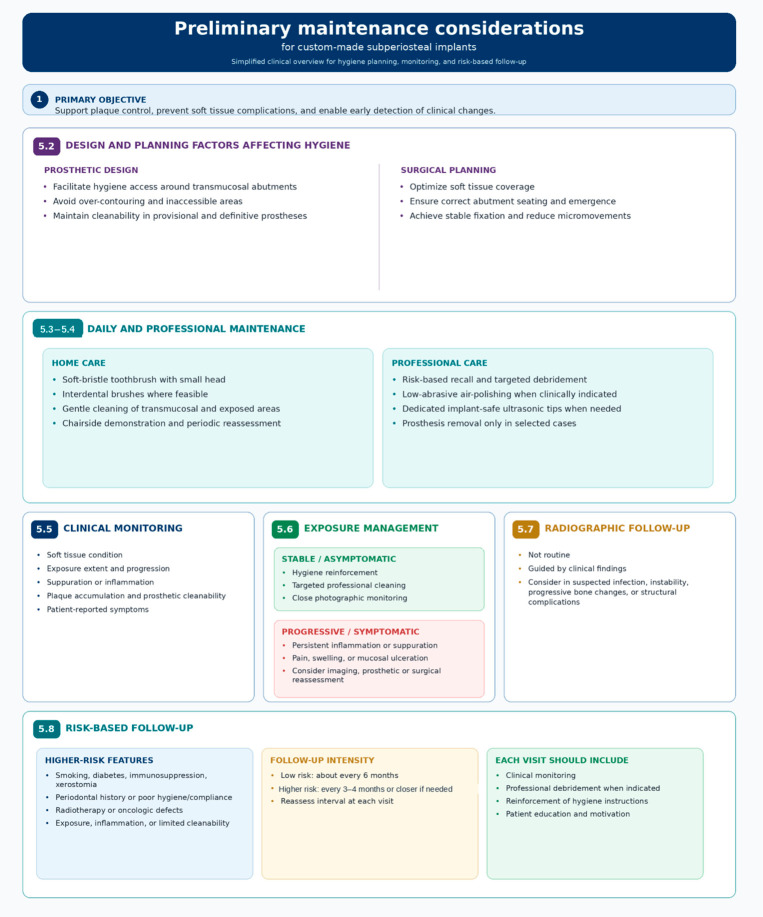
Evidence-informed maintenance approach for patients rehabilitated with custom-made subperiosteal implants.

The aim is to promote structured clinical observation, effective plaque control, patient education, and early identification of changes requiring closer follow-up or additional intervention (Figure 1).

### 5.2. Prosthetic Design and Surgical Planning Considerations for Hygiene Maintenance

The prosthetic design plays a critical role in the long-term maintenance of subperiosteal implant rehabilitations and should be considered an integral component of the overall hygiene strategy rather than a purely restorative element. In this context, the prosthesis is not a passive structure but actively influences the patient’s ability to perform adequate plaque control and, consequently, the long-term biological stability of the implant [76,77]. Given the complexity of these frameworks and the presence of multiple transmucosal components, the prosthesis should ideally be designed to facilitate plaque control and allow adequate access for both patient-performed and professional hygiene procedures [76,77,78]. In particular, the creation of dedicated hygiene access pathways at the level of the transmucosal abutments should be considered whenever prosthetically and surgically feasible. These access channels should ideally allow the use of simple and reproducible hygiene aids, particularly interdental brushes, enabling the patient to reach otherwise inaccessible areas and improve plaque control.

From a clinical standpoint, the absence of such access features may significantly increase the risk of biofilm retention, especially in concave regions or beneath prosthetic frameworks, where mechanical cleaning is inherently more difficult [76,77,78]. For this reason, prosthetic contours should be carefully designed to avoid over-contouring and to ensure adequate visibility and accessibility of critical zones during both home care and professional maintenance.

An important distinction should be made between provisional and definitive prosthetic phases. The provisional prosthesis should be intentionally designed to leave a sufficiently wide space between the prosthetic structure and the underlying tissues, allowing easy access for hygiene procedures during the early healing phase [79]. This facilitates patient adaptation to oral hygiene maneuvers and enables more effective plaque control in a period characterized by increased biological vulnerability. In contrast, the definitive prosthesis should maintain the same hygienic principles while optimizing functional and aesthetic outcomes, preserving access for cleaning without compromising structural integrity or patient comfort.

In addition to prosthetic considerations, surgical planning plays a fundamental role in determining the long-term maintainability of subperiosteal implants. Given the custom-made nature of these devices, implant design and positioning must be closely aligned with both prosthetic and hygiene requirements from the earliest stages of treatment planning. In particular, the presence of neighboring natural teeth and reduced distances between prosthetic coupling elements have been associated with a higher risk of complications in complex patient-specific subperiosteal implant rehabilitations [80]. Although these findings derive from a specific implant concept and should not be generalized without caution, they support the importance of careful spatial planning of transmucosal components, prosthetic access areas, and coupling-element distribution during the design phase. Furthermore, the number and distribution of transmucosal abutments should also be considered from a maintenance perspective. A recent systematic review on digitally constructed subperiosteal implants suggested that limiting the number of transmucosal abutments may help reduce bacterial colonization, with four abutments being proposed as a favorable configuration in full-arch rehabilitations [81]. However, this concept should be balanced against biomechanical stability, prosthetic support, implant extension, and patient-specific anatomical conditions. Therefore, abutment number should not be determined solely by hygiene considerations, but rather through an integrated prosthetic, biomechanical, and biological assessment.

Particular attention should be paid to the management of soft tissues. Achieving adequate vestibular coverage of the implant framework is essential to reduce exposure risk and improve long-term tissue stability. This may be facilitated by performing slightly palatal or lingual incisions in order to preserve and reposition keratinized mucosa, or, when necessary, by reinforcing soft tissues using techniques such as membrane application or the use of the buccal fat pad. These approaches contribute to creating a more stable and resistant soft tissue environment around transmucosal components [1,2,43,82].

Equally important is the precise preparation of the crestal sites for the transmucosal abutments. The seating of these components must be carefully planned and executed, as both under-preparation and over-preparation may lead to clinically relevant complications [1,2,43,83]. An insufficiently prepared site may prevent complete seating of the implant framework, compromising fit and stability. Conversely, excessive preparation may create recessed areas beneath the abutments, favoring the accumulation of debris and inflammatory tissue and complicating hygiene procedures.

Furthermore, accurate preoperative planning should include careful evaluation of abutment height in relation to soft tissue thickness. Abutments that are excessively prominent may hinder effective cleaning and increase plaque retention, whereas inadequate emergence may compromise accessibility and tissue health. A balanced approach is therefore required to ensure both functional and hygienic optimization [84,85].

Finally, adequate primary stability remains an important surgical objective [86]. Rigid fixation of the implant framework may help minimize micromovements at the implant–tissue interface, which could otherwise contribute to soft tissue irritation or inflammatory changes. Stable fixation may therefore support both mechanical performance and tissue stability.

### 5.3. Home Care Protocol

Effective home care represents a critical component of long-term maintenance, particularly in light of the structural complexity and limited accessibility of subperiosteal implant frameworks. For this reason, patients should receive individualized oral hygiene instructions tailored to the specific anatomical and prosthetic configuration, hygiene accessibility, manual dexterity, and ability to perform effective plaque control.

Home-care measures should prioritize simplicity, safety, and reproducibility. Mechanical plaque control should primarily rely on soft-bristle toothbrushes with small heads, allowing atraumatic cleaning of prosthetic margins and exposed or partially exposed components. Interdental brushes may represent the main adjunctive tool when adequate access is available, as their use may be more reproducible than that of more technique-sensitive devices in complex prosthetic rehabilitations. Single-tuft brushes may be considered as adjunctive aids in selected areas, but should not be regarded as first-line instruments for all patients.

The selection of home-care devices should therefore be individualized rather than standardized. In particular, hygiene aids requiring greater manual dexterity should be recommended cautiously and only when the patient demonstrates the ability to use them safely and effectively around the prosthetic components. Special attention should be directed toward the implant–mucosa interface, prosthetic margins, and any exposed implant areas, which represent critical zones for biofilm accumulation. Patients should be instructed to clean these areas carefully, balancing effective plaque disruption with the need to avoid soft tissue trauma.

The selected aids should be demonstrated chairside and periodically reassessed according to plaque control and tissue tolerance [87,88].

### 5.4. Professional Hygiene Protocol

Professional maintenance should be adapted to prosthetic complexity, accessibility of the transmucosal components, tissue conditions, and the presence of plaque-retentive areas or implant exposure [89,90]. The objective is to disrupt biofilm while minimizing trauma to soft tissues and implant surfaces. Recall frequency is addressed separately in the risk-based maintenance section.

Non-invasive instrumentation should be preferred in order to minimize tissue trauma and preserve implant surfaces. Air-polishing with low-abrasive powders, such as glycine or erythritol, may represent a useful minimally invasive approach for biofilm disruption when clinically indicated, particularly in areas where tissue conditions and access allow its safe use. Dedicated ultrasonic tips designed for implant maintenance may also facilitate targeted biofilm and calculus removal in areas with limited accessibility. During professional hygiene sessions, particular attention should be paid to transmucosal components, the implant–mucosa interface, exposed implant surfaces, and prosthetic margins. Areas characterized by limited accessibility or complex geometry require careful and targeted debridement, as they are more prone to plaque accumulation.

Periodic prosthesis removal may be considered in selected cases in order to facilitate inspection and debridement of subprosthetic areas that are difficult to access during routine maintenance procedures [60,91,92]. However, this approach should not be regarded as mandatory in all patients. In well-designed rehabilitations with adequate hygiene accessibility and stable clinical conditions, maintenance may often be effectively performed without routine prosthesis disassembly. The decision to remove the prosthesis should therefore be individualized according to prosthetic configuration, patient hygiene performance, and the presence of clinical signs such as inflammation, biofilm accumulation, or implant exposure. When performed, prosthesis removal may also provide an opportunity to assess prosthetic integrity, evaluate peri-implant tissues more thoroughly, and reinforce individualized hygiene instructions.

### 5.5. Clinical Monitoring

Clinical evaluation should be systematically performed at each follow-up visit, with particular focus on qualitative parameters rather than conventional quantitative measurements. At present, no validated diagnostic system exists for defining peri-implant health, soft tissue inflammation, exposure-related complications, or failure around custom-made subperiosteal implants. Therefore, monitoring should rely on standardized descriptive documentation rather than validated diagnostic thresholds.

Although no standardized qualitative assessment tool has been validated for subperiosteal implants, recent literature on additively manufactured subperiosteal jaw implants and related complex implant rehabilitations has increasingly emphasized the need to document soft tissue response, mucosal recession or dehiscence, mucositis-like inflammation, implant exposure, suppuration, hygiene accessibility, and patient-reported symptoms [41,47,58,68]. Similarly, research on peri-implant mucosal dehiscence and zygomatic implant maintenance suggests that qualitative parameters such as defect extension, progression over time, prosthetic cleanability, plaque retention, and associated symptoms may be clinically useful for longitudinal monitoring, although they remain non-validated in the specific context of subperiosteal implants [68].

Given the anatomical characteristics of subperiosteal implants, traditional peri-implant probing depth cannot be reliably applied and should not be considered a primary diagnostic tool. Instead, clinicians should descriptively document soft tissue condition, erythema, edema, bleeding tendency when gently evaluated, suppuration, plaque accumulation, prosthetic cleanability, extent and progression of implant exposure, and patient-reported symptoms such as pain or discomfort. Standardized intraoral photographs may be useful for longitudinal comparison of soft tissue conditions, exposed areas, and hygiene accessibility over time. These parameters should not be interpreted according to fixed thresholds, since validated diagnostic cut-offs are currently unavailable for subperiosteal implants.

### 5.6. Management of Implant Exposure

**Figure 2:**
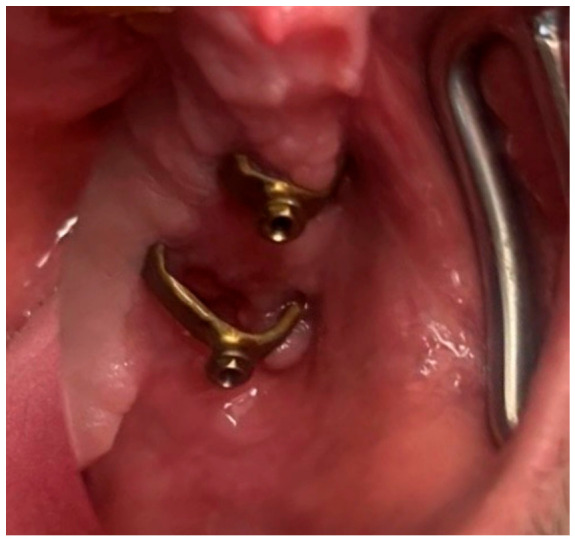
Clinical intraoral photograph showing mucosal dehiscence with exposure of the custom-made subperiosteal implant framework in the maxillary rehabilitation site.

Implant exposure represents a relatively frequent finding in subperiosteal implant rehabilitations and should be managed according to its clinical presentation [41,44,45,47] (Figure 2).

In the absence of symptoms, suppuration, implant mobility, or progressive soft tissue breakdown, a conservative approach may be considered [47]. This may include reinforcement of local hygiene measures, targeted professional cleaning of the exposed area, elimination of plaque-retentive prosthetic factors when feasible, and close photographic monitoring over time. Stable, asymptomatic exposure should not automatically be interpreted as treatment failure, because the prognostic significance of limited exposure remains uncertain in the absence of longitudinal evidence.

Transition from conservative management to surgical or prosthetic intervention should be considered when exposure is progressive, when low-grade inflammation persists despite optimized hygiene and repeated professional debridement, or when exposure is associated with suppuration, pain, recurrent swelling, mucosal ulceration, tissue fragility, or increasing plaque retention that cannot be controlled by the patient. This threshold for escalation may be lower in medically or oncologically complex patients, particularly in the presence of previous radiotherapy, xerostomia, diabetes, immunosuppression, or smoking, because these factors may impair mucosal healing and increase susceptibility to persistent inflammation or infection.

Additional warning signs include suspected implant instability, recurrent soft tissue breakdown over fixation areas or transmucosal components, and prosthetic designs that prevent effective access to the exposed framework. In these situations, further diagnostic evaluation, including imaging when clinically indicated, may be required to assess deep infection, bone changes, mechanical complications, or fixation-related problems.

Possible interventions may range from prosthetic modification to improve cleanability, local soft tissue management, surgical debridement, soft tissue coverage procedures, or, in selected cases, partial or complete implant removal. The choice of intervention should be individualized according to the extent and progression of exposure, presence of infection, implant stability, prosthetic accessibility, patient comorbidities, previous radiotherapy, and the functional relevance of the exposed component. Because validated exposure grading systems for subperiosteal implants are not currently available, these criteria should be regarded as pragmatic clinical indicators rather than standardized treatment thresholds.

### 5.7. Radiological Follow-Up

Radiological follow-up in subperiosteal implants differs fundamentally from that of endosseous implants. In osseointegrated systems, radiographic assessment represents a cornerstone of long-term monitoring, as marginal bone loss is a well-established surrogate marker of implant health and stability [93,94,95].

In contrast, subperiosteal implants do not rely on a circumferential endosseous bone–implant interface, and conventional radiographic parameters such as crestal bone-level changes are therefore not directly applicable. Routine radiographic follow-up should not be considered mandatory in the same manner as for endosseous implants [1,2,86]. Instead, imaging may be reserved for specific clinical indications, including suspected deep infection, unexplained pain, implant instability, progressive bone changes, or potential mechanical complications.

### 5.8. Risk-Based Maintenance Strategy and Practical Monitoring Domains

A personalized, risk-based approach may help structure maintenance planning for patients rehabilitated with subperiosteal implants. Several patient-related factors may influence the individual risk profile, including smoking, diabetes, immunosuppression, history of periodontal disease, xerostomia, previous radiotherapy, oncologic defects, oral hygiene effectiveness, patient compliance, and manual dexterity. These factors may impair soft tissue healing, reduce plaque-control capacity, increase susceptibility to inflammation or infection, and complicate long-term maintenance. Prosthetic and implant-related factors should also be considered, including the presence of implant exposure, limited hygiene accessibility, complex subprosthetic geometries, and the cleanability of the prosthetic rehabilitation. Practical monitoring domains that may be considered during follow-up are summarized in Table 2. These items are not validated diagnostic criteria, but pragmatic clinical descriptors that may support structured documentation and longitudinal comparison until dedicated diagnostic systems are developed.

Within this approach, the frequency of clinical evaluations and professional hygiene sessions should be tailored rather than predetermined according to fixed intervals. Although evidence-based recall intervals cannot currently be recommended for subperiosteal implants, 6-month reviews may be considered for clinically stable low-risk patients with good hygiene access, whereas 3–4-month or shorter individualized recalls may be considered for patients with progressive exposure, recurrent inflammation, poor plaque control, limited manual dexterity, history of periodontitis, previous radiotherapy, or prosthetic designs with limited cleanability. The interval should be reassessed at each visit according to clinical stability, tissue condition, hygiene performance, and patient compliance.

**Figure 3:**
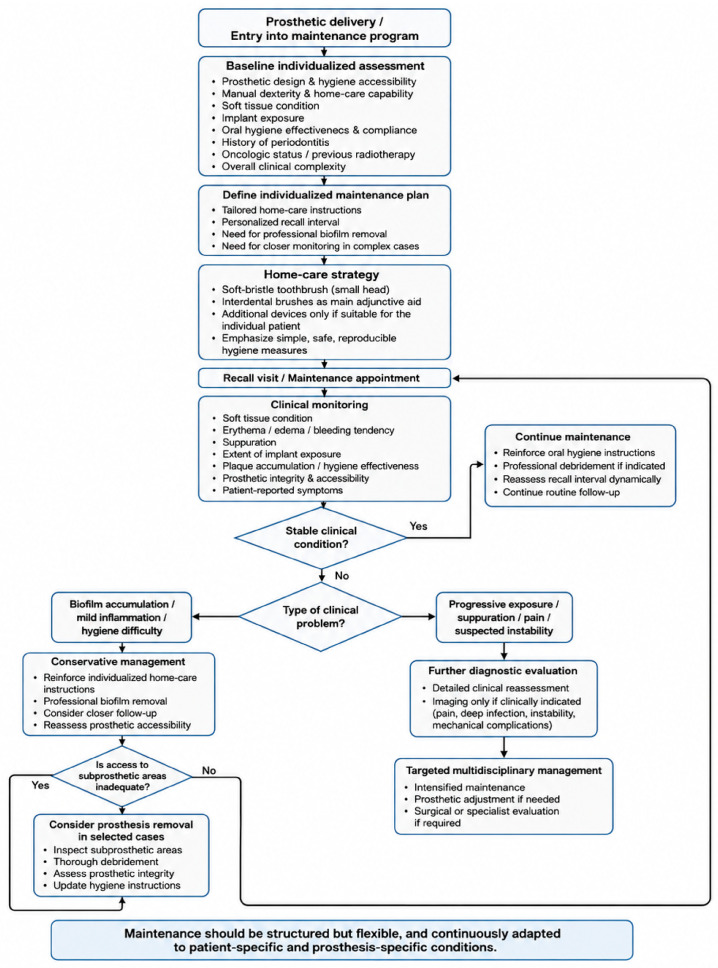
Evidence-informed clinical maintenance flowchart for patients rehabilitated with custom-made subperiosteal implants.

The content of each maintenance visit should also be individualized. Some appointments may primarily focus on clinical monitoring, motivation, and reinforcement of home-care instructions, whereas others may require professional biofilm removal or, in selected cases, prosthesis removal for more thorough inspection and debridement. Importantly, each maintenance appointment should not be limited to passive observation, but should include active patient education and motivation. Reinforcement of oral hygiene techniques should be considered an integral component of every visit, particularly in the early phases following prosthetic delivery and in patients demonstrating suboptimal plaque control (Figure 3).

## 6. Limitations

The present study presents several limitations that should be acknowledged. First, the proposed maintenance approach is not supported by direct high-level evidence specifically addressing subperiosteal implants. The available literature on these devices remains limited, consisting predominantly of case reports, technical notes, and small case series, with a primary focus on surgical aspects rather than long-term maintenance strategies.

Second, the evidence used to develop the proposed clinical considerations has been largely extrapolated from related fields, including endosseous implantology, supportive periodontal therapy, full-arch rehabilitations, and zygomatic implant maintenance. Although this approach is justified by shared biological and clinical principles, it inevitably introduces a degree of indirectness that may limit the generalizability of the proposed principles.

Furthermore, the heterogeneity of patient populations, implant designs, and clinical indications represents an additional limitation. Subperiosteal implants are typically used in complex clinical scenarios, including severe atrophy and post-oncological defects, which are associated with variable systemic conditions, soft tissue quality, and functional demands. These factors may influence both the risk profile and the response to maintenance strategies.

Another limitation is the potential for interpretive bias. The contemporary literature on custom-made subperiosteal implants remains limited and is produced by a relatively small number of recurring research groups. This reflects the emerging nature of the field, but may reduce the balance and external validity of available interpretations. In addition, some of the clinical considerations discussed in this review are informed by the authors’ experience with the follow-up of patients rehabilitated with custom-made subperiosteal implants. In the authors’ institution, patients were generally followed according to a structured maintenance strategy including periodic clinical evaluation, reinforcement of oral hygiene instructions, professional plaque control, photographic documentation when clinically indicated, and closer follow-up in cases of exposure, inflammation, poor hygiene accessibility, or oncologic risk factors. However, this approach was not applied as a prospectively registered or externally validated protocol, and no unpublished patient-level data, predefined outcome analysis, or comparative evaluation is presented in the present review. Therefore, the authors’ clinical experience should be regarded only as contextual background and not as evidence supporting validated recommendations.

Finally, the literature search was broad but not systematic. Although multiple databases and reference lists were screened, no formal PRISMA-based selection process, duplicate independent screening, or risk-of-bias assessment was performed. As a result, selection bias cannot be excluded. This limitation is particularly relevant in an emerging field such as subperiosteal implantology, where terminology is heterogeneous and relevant reports may be indexed inconsistently.

## 7. Future Directions

The growing clinical interest in custom-made subperiosteal implants [96,97,98,99] highlights the urgent need for dedicated research focused on their long-term maintenance. Future studies should aim to evaluate the effectiveness of structured maintenance strategies in this specific patient population through prospective clinical trials and multicenter cohort studies. Future research should also stratify outcomes according to patient-related risk factors, including smoking, diabetes, immunosuppression, xerostomia, previous radiotherapy, and oncologic status, as these variables may substantially influence maintenance needs and complication risk.

A major research priority is the development and validation of diagnostic criteria specifically designed for subperiosteal implants. Such criteria should include standardized assessment of soft tissue inflammation, suppuration, exposure size and progression, prosthetic cleanability, patient-reported symptoms, mechanical stability, and imaging findings when clinically indicated. Prospective validation is required before these parameters can be used as formal diagnostic or prognostic tools.

Further investigation is also warranted to better understand the relationship between prosthetic design, hygiene accessibility, and long-term clinical outcomes. Studies evaluating the impact of hygiene-oriented prosthetic features, including access channels and subprosthetic space configuration, could provide valuable insights for optimizing both surgical and restorative planning.

Future studies should also investigate implant-related design and manufacturing variables that may influence both biological and mechanical outcomes. These include the optimal thickness of the subperiosteal framework, the balance between rigidity and prosthetic bulk, and the potential role of alternative materials or surface characteristics in reducing plaque retention and bacterial colonization. In addition, hybrid additive–subtractive manufacturing workflows, combining the geometric flexibility of additive manufacturing with the surface refinement and precision of milling, may represent a promising area for future research. However, their potential advantages over conventional milling or additive manufacturing alone require dedicated mechanical, microbiological, and clinical validation before any recommendation can be made.

Moreover, future studies should also investigate the effectiveness, safety, and patient acceptability of different home-care devices in subperiosteal implant rehabilitations, with particular attention to ease of use, risk of improper use, and reproducibility in daily practice. The integration of digital tools for monitoring, such as intraoral scanning and photographic documentation, may further enhance longitudinal assessment and early detection of complications.

Finally, expert consensus initiatives, such as Delphi processes, may represent a valuable step toward the development of standardized clinical guidelines, facilitating broader adoption and improving reproducibility of maintenance strategies across different clinical settings.

## 8. Conclusions

Custom-made subperiosteal implants represent a valuable and increasingly utilized option for the rehabilitation of patients with severe maxillofacial atrophy and complex reconstructive needs. However, their unique anatomical, biological, and biomechanical characteristics differentiate them substantially from conventional endosseous implants, particularly with regard to long-term maintenance requirements.

The present narrative review highlights the absence of dedicated maintenance guidelines or validated diagnostic and therapeutic strategies for custom-made subperiosteal implants. It also emphasizes that conventional peri-implant maintenance protocols cannot be directly transferred to these rehabilitations because of differences in anatomy, tissue interface, biomechanics, prosthetic accessibility, and clinical monitoring parameters.

Within these limitations, the proposed clinical considerations may provide a preliminary structure for maintenance planning, with emphasis on plaque control, descriptive clinical monitoring, patient education, prosthetic cleanability, and individualized follow-up. Until validated diagnostic systems are available, clinical monitoring should rely on structured descriptive assessment rather than conventional endosseous implant parameters.

These considerations should be regarded as hypothesis-generating and require validation through prospective studies, multicenter registries, and expert consensus initiatives before definitive maintenance approaches can be recommended.

## Figures and Tables

**Table 1:** Source of evidence, degree of indirectness, and possible clinical implications for maintenance of custom-made subperiosteal implants.

Clinical Issue	Direct Evidence in Subperiosteal Implants	Indirect Evidence Source	Degree of Indirectness	Possible Clinical Implication
Biofilm control	Very limited	Endosseous implants, full-arch prostheses	High	Professional and home-care biofilm control may be clinically reasonable, but specific protocols are not validated
Recall intervals	Absent	Supportive periodontal and peri-implant therapy	High	Recall should be individualized according to risk profile rather than fixed
Prosthetic cleanability	Limited	Full-arch implant prostheses	Moderate/high	Hygiene-oriented prosthetic design may improve access and plaque control
Implant exposure	Limited case series and technical reports	Reconstructive hardware, zygomatic implants	Moderate/high	Asymptomatic exposure may be monitored conservatively, whereas progressive or infected exposure requires reassessment
Probing depth	No validation	Anatomical rationale	High	Probing depth should not be used as a primary diagnostic criterion
Radiographic monitoring	No validated markers	Endosseous implant follow-up	High	Imaging should be symptom-driven or complication-driven rather than based on routine crestal bone-level assessment
Risk of over-translation	No direct validation	Endosseous implants, periodontal maintenance, zygomatic implants	High	Indirect evidence should be used to identify general principles only; diagnostic thresholds and maintenance protocols should not be transferred without adaptation

**Table 2:** Practical monitoring domains and possible clinical actions in patients rehabilitated with custom-made subperiosteal implants.

Domain	Clinical Finding	Possible Interpretation	Possible Clinical Action
Soft tissues	Erythema, edema, mucosal fragility	Local inflammatory response or inadequate plaque control	Reinforce home care; perform targeted professional debridement; shorten recall interval
Exposure	Stable, asymptomatic exposure	Potential plaque-retentive area without evident progression	Conservative monitoring; targeted hygiene instruction; photographic documentation
Exposure	Progressive exposure, persistent low-grade inflammation, suppuration, pain, recurrent swelling, or mucosal ulceration	Possible biological complication, tissue breakdown, or uncontrolled plaque-retentive area	Shorten recall interval; reassess prosthetic cleanability; consider imaging if indicated; surgical/prosthetic intervention if progression persists
Hygiene access	Plaque or debris retention beneath the prosthesis	Insufficient access or ineffective home care	Modify hygiene aids; consider professional debridement; consider prosthesis removal in selected cases
Symptoms	Recurrent swelling, pain, discomfort, instability sensation	Possible inflammation, mechanical issue, or deep complication	Detailed clinical evaluation; imaging when persistent or unexplained
Prosthesis	Overcontouring, inaccessible embrasures, poor cleanability	Design-related maintenance risk	Reinforce professional maintenance; consider prosthetic modification if feasible
Patient factors	Smoking, diabetes, immunosuppression, xerostomia, previous radiotherapy, oncologic defects, poor compliance, reduced dexterity, history of periodontitis	Increased biological and maintenance-related risk	Shorter recall interval; intensified motivation; tailored home-care aids; closer monitoring of exposure and inflammation
Longitudinal change	Increase in exposure size, worsening erythema, recurrent swelling, or reduced cleanability over time	Possible progression of soft tissue instability or maintenance failure	Compare with previous photographs; shorten recall interval; consider imaging or surgical/prosthetic reassessment if progression persists

## Data Availability

No new data were created or analyzed in this study. Data sharing is not applicable to this article.

## Supplementary Materials

The following supporting information can be downloaded at: https://www.mdpi.com/article/10.3390/jcm15114333/s1, Table S1: Search strategy.
