# Supplementary material for: Maintenance of Custom-Made Subperiosteal Implants: A Narrative Review of Indirect Evidence and Preliminary Clinical Considerations
Source: J Clin Med. 2026 Jun 3;15(11):4333. doi: 10.3390/jcm15114333 (PMC13257507; doi:10.3390/jcm15114333)
Supplement: Supplementary file 1 [file jcm-15-04333-s001.zip › jcm-4332482-supplementary.pdf]

Supplementary Table S1. Search strategy

Database	Search String
PubMed	(“subperiosteal implants” OR “juxtaosseous implants” OR “custom-made implants” OR “patient-specific implants”) AND (“peri-implant maintenance” OR “supportive therapy” OR “implant maintenance” OR “oral hygiene” OR “recall program”)
Scopus	TITLE-ABS-KEY (“subperiosteal implants” OR “juxtaosseous implants” OR “custom-made implants” OR “patient-specific implants”) AND TITLE-ABS-KEY (“peri-implant maintenance” OR “supportive therapy” OR “implant maintenance” OR “oral hygiene” OR “recall program”)
Web of Science	TS=(“subperiosteal implants” OR “juxtaosseous implants” OR “custom-made implants” OR “patient-specific implants”) AND TS=(“peri-implant maintenance” OR “supportive therapy” OR “implant maintenance” OR “oral hygiene” OR “recall program”)
